# The potential for artificial intelligence to transform healthcare: perspectives from international health leaders

**DOI:** 10.1038/s41746-024-01097-6

**Published:** 2024-04-09

**Authors:** Christina Silcox, Eyal Zimlichmann, Katie Huber, Neil Rowen, Robert Saunders, Mark McClellan, Charles N. Kahn, Claudia A. Salzberg, David W. Bates

**Affiliations:** 1 Duke-Margolis Institute for Health Policy, Duke University, Washington, DC, USA &, Durham, NC USA; 2 Sheba Medical Center, Ramat Gan, Israel; 3 Future of Health, Washington, DC USA; 4 Federation of American Hospitals, Washington, DC USA; 5 Division of General Internal Medicine, Brigham and Women’s Hospital, Boston, MA USA; 6 Harvard Medical School, Boston, MA USA; 7 Department of Health Policy and Management, Harvard T. H. Chan School of Public Health, Boston, MA USA

**Keywords:** Health policy, Health services

## Abstract

Artificial intelligence (AI) has the potential to transform care delivery by improving health outcomes, patient safety, and the affordability and accessibility of high-quality care. AI will be critical to building an infrastructure capable of caring for an increasingly aging population, utilizing an ever-increasing knowledge of disease and options for precision treatments, and combatting workforce shortages and burnout of medical professionals. However, we are not currently on track to create this future. This is in part because the health data needed to train, test, use, and surveil these tools are generally neither standardized nor accessible. There is also universal concern about the ability to monitor health AI tools for changes in performance as they are implemented in new places, used with diverse populations, and over time as health data may change. The Future of Health (FOH), an international community of senior health care leaders, collaborated with the Duke-Margolis Institute for Health Policy to conduct a literature review, expert convening, and consensus-building exercise around this topic. This commentary summarizes the four priority action areas and recommendations for health care organizations and policymakers across the globe that FOH members identified as important for fully realizing AI’s potential in health care: improving data quality to power AI, building infrastructure to encourage efficient and trustworthy development and evaluations, sharing data for better AI, and providing incentives to accelerate the progress and impact of AI.

## Introduction

Artificial intelligence (AI), supported by timely and accurate data and evidence, has the potential to transform health care delivery by improving health outcomes, patient safety, and the affordability and accessibility of high-quality care^1,2^. AI integration is critical to building an infrastructure capable of caring for an increasingly aging population, utilizing an ever-increasing knowledge of disease and options for precision treatments, and combatting workforce shortages and burnout of medical professionals. However, we are not currently on track to create this future. This is in part because the health data needed to train, test, use, and surveil these tools are generally neither standardized nor accessible. This is true across the international community, although there is variable progress within individual countries. There is also universal concern about monitoring health AI tools for changes in performance as they are implemented in new places, used with diverse populations, and over time as health data may change.

The Future of Health (FOH) is an international community of senior health care leaders representing health systems, health policy, health care technology, venture funding, insurance, and risk management. FOH collaborated with the Duke-Margolis Institute for Health Policy to conduct a literature review, expert convening, and consensus-building exercise. In total, 46 senior health care leaders were engaged in this work, from eleven countries in Europe, North America, Africa, Asia, and Australia. This commentary summarizes the four priority action areas and recommendations for health care organizations and policymakers that FOH members identified as important for fully realizing AI’s potential in health care: improving data quality to power AI, building infrastructure to encourage efficient and trustworthy development and evaluations, sharing data for better AI, and providing incentives to accelerate the progress and impact of AI.

### Powering AI through high-quality data

*“Going forward, data are going to be the most valuable commodity in health care. Organizations need robust plans about how to mobilize and use their data.”*

AI algorithms will only perform as well as the accuracy and completeness of key underlying data, and data quality is dependent on actions and workflows that encourage trust.

To begin to improve data quality, FOH members agreed that an initial priority is identifying and assuring reliable availability of high-priority data elements for promising AI applications: those with the most predictive value, those of the highest value to patients, and those most important for analyses of performance, including subgroup analyses to detect bias.

Leaders should also advocate for aligned policy incentives to improve the availability and reliability of these priority data elements. There are several examples of efforts across the world to identify and standardize high-priority data elements for AI applications and beyond, such as the multinational project STANDING Together, which is developing standards to improve the quality and representativeness of data used to build and test AI tools^3^.

Policy incentives that would further encourage high-quality data collection include (1) aligned payment incentives for measures of health care quality and safety, and ensuring the reliability of the underlying data, and (2) quality measures and performance standards focused on the reliability, completeness, and timeliness of collection and sharing of high-priority data itself.

### Trust and verify

*“Your AI algorithms are only going to be as good as the data and the real-world evidence used to validate them, and the data are only going to be as good as the trust and privacy and supporting policies.”*

FOH members stressed the importance of showing that AI tools are both effective and safe within their specific patient populations.

This is a particular challenge with AI tools, whose performance can differ dramatically across sites and over time, as health data patterns and population characteristics vary. For example, several studies of the Epic Sepsis Model found both location-based differences in performance and degradation in performance over time due to data drift^4,5^. However, real-world evaluations are often much more difficult for algorithms that are used for longer-term predictions, or to avert long-term complications from occurring, particularly in the absence of connected, longitudinal data infrastructure. As such, health systems must prioritize implementing data standards and data infrastructure that can facilitate the retraining or tuning of algorithms, test for local performance and bias, and ensure scalability across the organization and longer-term applications^6^.

There are efforts to help leaders and health systems develop consensus-based evaluation techniques and infrastructure for AI tools, including HealthAI: The Global Agency for Responsible AI in Health, which aims to build and certify validation mechanisms for nations and regions to adopt; and the Coalition for Health AI (CHAI), which recently announced plans to build a US-wide health AI assurance labs network^7,8^. These efforts, if successful, will assist manufacturers and health systems in complying with new laws, rules, and regulations being proposed and released that seek to ensure AI tools are trustworthy, such as the EU AI Act and the 2023 US Executive Order on AI.

### Sharing data for better AI

*“Underlying these challenges is the investment required to standardize business processes so that you actually get data that’s usable between institutions and even within an institution.”*

While high-quality internal data may enable some types of AI-tool development and testing, this is insufficient to power and evaluate all AI applications. To build truly effective AI-enabled predictive software for clinical care and predictive supports, data often need to be interoperable across health systems to build a diverse picture of patients’ health across geographies, and reliably shared.

FOH members recommended that health care leaders work with researchers and policymakers to connect detailed encounter data with longitudinal outcomes, and pilot opportunities across diverse populations and systems to help assure valid outcome evaluations as well as address potential confounding and population subgroup differences—the ability to aggregate data is a clear rate-limiting step. The South African National Digital Health Strategy outlined interventions to improve the adoption of digital technologies while complying with the 2013 Protection of Personal Information Act^9^. Although challenges remain, the country has made progress on multiple fronts, including building out a Health Patient Registration System as a first step towards a portable, longitudinal patient record system and releasing a Health Normative Standards Framework to improve data flow across institutional and geographic boundaries^10^.

Leaders should adopt policies in their organizations, and encourage adoption in their province and country, that simplify data governance and sharing while providing appropriate privacy protections – including building foundations of trust with patients and the public as previously discussed. Privacy-preserving innovations include ways to “share” data without movement from protected systems using approaches like federated analyses, data sandboxes, or synthetic data. In addition to exploring privacy-preserving approaches to data sharing, countries and health systems may need to consider broad and dynamic approaches to consent^11,12^. As we look to a future where a patient may have thousands of algorithms churning away at their data, efforts to improve data quality and sharing should include enabling patients’ access to and engagement with their own data to encourage them to actively partner in their health and provide transparency on how their data are being used to improve health care. For example, the Understanding Patient Data program in the United Kingdom produces research and resources to explain how the National Health Service uses patients’ data^13^. Community engagement efforts can further assist with these efforts by building trust and expanding understanding.

FOH members also stressed the importance of timely data access. Health systems should work together to establish re-usable governance and privacy frameworks that allow stakeholders to clearly understand what data will be shared and how it will be protected to reduce the time needed for data use agreements. Trusted third-party data coordinating centers could also be used to set up “precertification” systems around data quality, testing, and cybersecurity to support health organizations with appropriate data stewardship to form partnerships and access data rapidly.

### Incentivizing progress for AI impact

*“Unless it’s tied to some kind of compensation to the organization, the drive to help implement those tools and overcome that risk aversion is going to be very high… I do think that business driver needs to be there.”*

AI tools and data quality initiatives have not moved as quickly in health care due to the lack of direct payment, and often, misalignment of financial incentives and supports for high-quality data collection and predictive analytics. This affects both the ability to purchase and safely implement commercial AI products as well as the development of “homegrown” AI tools.

FOH members recommended that leaders should advocate for paying for value in health – quality, safety, better health, and lower costs for patients. This better aligns the financial incentives for accelerating the development, evaluation, and adoption of AI as well as other tools designed to either keep patients healthy or quickly diagnose and treat them with the most effective therapies when they do become ill. Effective personalized health care requires high-quality, standardized, interoperable datasets from diverse sources^14^. Within value-based payments themselves, data are critical to measuring quality of care and patient outcomes, adjusted or contextualized for factors outside of clinical control. Value-based payments therefore align incentives for (1) high-quality data collection and trusted use, (2) building effective AI tools, and (3) ensuring that those tools are improving patient outcomes and/or health system operations.

## Conclusion

Data have become the most valuable commodity in health care, but questions remain about whether there will be an AI “revolution” or “evolution” in health care delivery. Early AI applications in certain clinical areas have been promising, but more advanced AI tools will require higher quality, real-world data that is interoperable and secure. The steps health care organization leaders and policymakers take in the coming years, starting with short-term opportunities to develop meaningful AI applications that achieve measurable improvements in outcomes and costs, will be critical in enabling this future that can improve health outcomes, safety, affordability, and equity.

## Data Availability

Data sharing is not applicable to this article as no datasets were generated or analyzed during the current study.
